# Endoscopic negative pressure therapy of the upper gastrointestinal tract

**DOI:** 10.1007/s00104-018-0727-x

**Published:** 2018-11-19

**Authors:** G. Loske

**Affiliations:** Department of General, Visceral, Thoracic and Vascular Surgery, Katholisches Marienkrankenhaus Hamburg gGmbH, Alfredstr. 9, 22087 Hamburg, Germany

**Keywords:** Endoscopic vacuum therapy, Esophagus, Drainage, Anastomotic leakage, Perforation, Endoskopische Vakuumtherapie, Ösophagus, Drainage, Anastomoseninsuffizienz, Perforation

## Abstract

Endoscopic negative pressure therapy (ENPT) has been adapted for upper gastrointestinal tract applications. More than 400 patients have already been treated with ENPT due to transmural defects in the upper gastrointestinal tract, with a success rate of 87%. The greatest experience exists for the treatment of anastomotic leakages and perforations of the esophagus. The ENPT is also used in the duodenum, pancreas and for complications after bariatric surgery. There are new indications that go beyond treatment in complication management. Innovative drainage types and endoscopic techniques have been developed that broaden the spectrum of applications. The aim of this article is to give an overview of the current status of ENPT in the upper gastrointestinal tract.

## Zusammenfassung

Die endoskopische Unterdrucktherapie (EUT) wurde für Anwendungen im oberen Gastrointestinaltrakt (OGI) adaptiert. Bereits mehr als 400 Patienten wurden aufgrund transmuraler Intestinaldefekte im OGI behandelt, die Erfolgsrate liegt bei 87 %. Die größte Erfahrung besteht für die Therapie von Anastomoseninsuffizienzen und Perforationen am Ösophagus. Die EUT wird auch am Duodenum, Pankreas und bei Komplikationen nach bariatrischer Chirurgie angewandt. Es bestehen neue Indikationen, die über die Behandlungen im Komplikationsmanagement hinausgehen. Innovative Drainagetypen und endoskopische Techniken erweitern das breite Anwendungsspektrum. Das Ziel der Arbeit ist es, einen Überblick über den derzeitigen Stand der EUT im oberen Gastrointestinaltrakt zu geben.

## Background

We used endoscopic negative pressure therapy (ENPT) in the upper gastrointestinal tract for the very first time in 2006. We adapted the method previously used in the rectum to treat an anastomotic leak after gastrectomy [1–3]. The anastomotic defect healed without the need for surgical revision. A small case series was then presented at the “Viszeralmedizin” conference in 2007 at which Wallstabe et al. also reported on the successful use of ENPT in a very complicated case involving the esophagus [4]. In 2010 we published the first original paper on ENPT in the esophagus with a case series consisting of ten anastomotic leaks and perforations with a 90% success rate [5].

Based on this experience, ENPT of the upper gastrointestinal tract was adopted by several German surgical working groups and the positive results were confirmed [6, 7]. To date, ENPT of the upper gastrointestinal tract has been used on more than 420 patients worldwide with an 87% success rate ([8–25]; Table 1). In the vast majority of cases, it was performed on patients with transmural esophageal defects. A current study reports on the initial experience in pediatric patients [25]. In a smaller number of cases, treatment also has been performed in the duodenum, in the pancreas, and for complications after bariatric surgery.

**Table 1:** Studies in which more than five patients were treated with ENPT in the upper gastrointestinal tract

Studies	Treatment site	Patients with ENPT (*n*)	Patients with successful ENPT (*n*)	Successful ENPT (%)
Brangwitz et al. [8]	E	32	27	84
Heits et al. [9]	E	10	9	90
Kühn et al. [10]	E	21	19	90
Laukötter et al. [11]	E	52	49	93
Locke et al. [12]	E	25	23	92
Möschler et al. [13]	E	10	7	70
Schniewind et al. [14]	E	17	15	88
Schorsch et al. [15]	E	35	32	91
Tan et al. [16]	E	12	10	83
Weidenhagen et al. [17]	E	6	6	100
Hwang et al. [18]	E	7	7	100
Loske et al. [19]	D	10	10	100
Oei et al. [20]	E	10	6	60
Pournaras et al. [21]	E, D, G	21	20	95
Bludau et al. [22]	E	77	60	78
Menico et al. [23]	E, B	39	36	92
Christogianni et al. [24]	B	21	18	85
Manfredi et al. [25]	E (in pediatric patients)	17	15	88
Total		422	369	87

*B* bariatric surgery, *D* duodenum, *E* esophagus, *ENPT* endoscopic negative pressure therapy,* G* stomach

To date, there is no clearly established terminology for the new treatment. In English, “endoscopic vacuum therapy” (EVT) has become the established term, but other terminology such as “EndoVac” and “E-Vac” is also used. In this article, the term “endoscopic negative pressure therapy” (ENPT) will be used, as the treatment strictly speaking does not involve the use of a vacuum but of negative pressure. A corresponding German term is “Endoskopische Unterdruck Therapie” (EUT).

## Principles of ENPT: defect closure and active drainage using negative pressure

ENPT is a further development of negative pressure therapy for external wound healing by secondary intention. This involves inserting large-pore polyurethane foam (PUF) pieces into the wound as open-pore drainage elements (OD; [26]). Once the wound is sealed with an occlusion film, a defined negative pressure is applied over a period of several days using an electronic negative pressure pump. The open-pore foam allows suction to be transmitted and exerted over the entire wound surface. The positive and useful effects of negative pressure are improved local perfusion, resolution of interstitial wound edema, suctioning of secretions, removal of slough, and debridement of the wound surface. Vital granulation tissue forms as the wound is cleaned and secondary wound healing can occur.

Endoscopy facilitates the intracorporeal application of negative pressure therapy along the natural orifices of the human body. Drainage tubes with an OD affixed at the distal end are advanced to the internal wound site via the nose or anus using various endoscopic techniques [27]. Suction is exerted where the OD is placed, and no further sealing is required. As with negative pressure therapy for superficial wounds, treatment is performed over a period of several days. Drain change intervals of 3–5 days have proven effective. If the clinical findings deteriorate and a malfunction is suspected, then early follow-up endoscopy is performed. Once suction is discontinued, the drain is removed by pulling on the drainage tube. Local wound healing is then endoscopically evaluated and, depending on the findings, continued, terminated, or the treatment procedure is changed. Assessment of wound healing requires surgical expertise.

## Intracavitary and intraluminal ENPT

There are two versions of ENPT depending on where the OD is placed: intracavitary and intraluminal ENPT [28, 29].

In intracavitary ENPT, the OD is introduced through the transmural intestinal defect into the extraluminal wound cavity. The wound cavity is emptied and continuously drained by applying negative pressure. It collapses with and around the OD. The intestinal defect also collapses around the drainage tube or the OD if the latter projects from the defect like “a cork”. This seals the defect opening and prevents further contamination by invasive pathological secretions.

In intraluminal ENPT, the OD is placed directly in the intestinal lumen. Drains with long, cylindrical ODs (up to 12 cm in length) are advantageous for treatment in the esophagus. These are placed in such a way that the defect comes to lie in the center of the OD, thereby ensuring that the defect zone is bridged along the oral–aboral axis. Once negative pressure has been applied, the esophageal lumen will collapse over and with the defect zone. Negative pressure temporarily induces therapeutic closure of the esophagus for as long as it is applied.

The defect is sealed and contamination is stopped immediately once suction takes hold. When intraluminal ENPT is applied in the stomach or duodenum, the drainage effect is often paramount. Continuous drainage leaves the stomach and duodenum “dry”.

The most important mechanisms of action of ENPT are the simultaneous closure of the defect and drainage in the wound area.

In 1926, M. Kirschner formulated these basic surgical principles for the treatment of peritonitis:

The treatment of open, suppurative peritonitis involves a) blocking the source of the infection, b) managing the exudate and cleaning the peritoneal cavity, c) draining the exudate and d) performing follow-up care [30].

## Materials: open-pore drains and electronic pumps

**Fig. 1:**
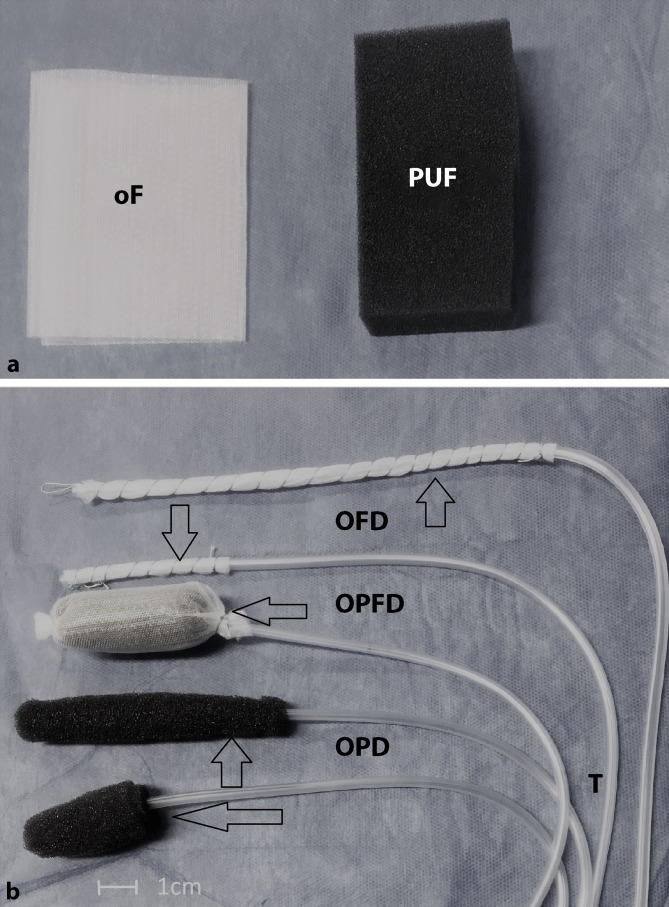
**a** Materials used to make open-pore drains for endoscopic negative pressure therapy (ENPT): double-layer open-pore film (*oF*) and open-pore polyurethane foam (*PUF*). **b** Different types of open-pore drains used for ENPT in the upper gastrointestinal tract. The distal end of a drainage tube (*T*) is wrapped with a drainage element: with open-pore polyurethane foam (*OPD*), with open-pore film (*OFD*), with OPD that is also wrapped with open-pore film (*OPFD*)

The material consists of negative pressure-resistant tubes that are up to 150 cm in length, with lateral perforations at the distal end. This distal section is wrapped with an OD, such as a large-pore PUF. Most of the reported applications used self-made drains (Fig. 1).

One open-pore PUF drain (OPD) is currently approved as a medical device for treatment in the esophagus and is commercially available (EsoSPONGE®, B. Braun Melsungen AG, Melsungen, Germany).

However, no electronic pump system has been approved to date for ENPT. In the upper gastrointestinal tract, we exclusively use electronic pumps that rapidly build up suction. Standard negative pressure of −125 mm Hg continuous suction has proven effective for all applications.

## New open-pore polyurethane foam drains

Several OPDs have been developed with different advantages. Short OPDs measuring only a few centimeters are used for intracavitary ENPT. Long OPDs measuring up to 12 cm and more in length can be used for intraluminal ENPT ([31]; Fig. 1). OPDs have been developed for placement using the pull-through technique in which the OD lies in the middle section of the drain [32, 33]. This considerably simplifies endoscopic placement in the presence of an enterocutaneous fistula. To allow for intestinal feeding during intraluminal ENPT, double-lumen OPDs have been developed with an additional jejunal feeding tube [34, 35].

## New open-pore film drains

Small-caliber, open-pore film drains (OFDs) have been developed using a very thin open-pore, double-layer, drainage film (Suprasorb® CNP Drainage Film, Lohmann & Rauscher International GmbH & Co, Rengsdorf, Germany), which is approved for negative pressure therapy of the abdomen [36]. The film is wrapped around the perforations in the tubes instead of the PUF (Fig. 1). These new drains have the advantage of a very small diameter of just a few millimeters, which facilitates their introduction through small openings and transnasal placement [37]. The PUF can also be wrapped with the film [33, 38]. These drains adhere less tightly to the wound bed under suction.

## ENPT in the esophagus

To date, the most extensive experience with ENPT in the upper gastrointestinal tract has been in the esophagus. The indication was first seen in the management of complications following esophageal resection with intrathoracic anastomosis. Even small transmural leaks induce mediastinitis [39]. The respiration-related intrathoracic fluctuations in negative pressure facilitate the extraluminal transport of even small amounts of secretion. Surgical trauma additionally promotes the rapid spread of infection. Other indications for ENPT include all other types of transmural esophageal injuries and therefore not only anastomotic, but also iatrogenic, spontaneous, and other leaks can be treated. One particular advantage of ENPT in the esophagus is that it allows for treatment of defects in any region, from the high cervical to the gastroesophageal junction.

Particularly good outcomes can be achieved in the treatment of iatrogenic perforations. We achieved a 100% healing rate in ten patients over a treatment period lasting just 5 days [40]. In a current overview of three studies with a total of 31 patients, all the patients were successfully treated with ENPT [41].

Five retrospective studies compared ENPT with treatment using covered, self-expanding metal stents (SEMS). An initial study compared the experience in pediatric patients [25] and demonstrated a significantly better outcome for ENPT. The other studies also demonstrated the superiority of ENPT [8, 14, 18, 42]. The Kiel working group showed that seriously ill patients in particular benefit from ENPT compared with surgical revision and stent procedures [14].

ENPT can be used “pre-emptively” for anastomosis protection

To date, several retrospective studies on ENPT have reported on more than 300 patients with esophageal defects of varying etiology [7–18, 20–23, 25]. Healing rates range from 60 to 100% (Table 1). A further case report described the successful use in an infant [43]. Reports of bleeding complications [11, 21] or serious complications in intracavitary ENPT are rare.

A further development in the esophagus is referred to as “pre-emptive ENPT”. If a suspicious anastomosis is found during follow-up endoscopy after esophageal resection, then intraluminal ENPT is performed even before a defect forms [44, 45]. Neumann et al. showed in a case series of eight patients that ENPT leads to healing in the presence of circumscribed anastomotic ischemia [46]. In two of the eight patients, a transmural anastomotic defect developed in course of pre-emptive treatment that could be exclusively treated with ENPT.

A further conceivable use of ENPT on the esophagus would be to promote anastomotic healing already during surgery. In this regard, a pilot study in a pig model is already available [47]. After abdominothoracic esophageal resection, an anastomotic defect was left, the anastomosis was bridged with an OPD during surgery, and intraluminal ENPT was initiated. The anastomosis had healed in all five animals after 5 days. The Munster study group is currently looking at whether ENPT can reduce the risk for the occurrence of anastomotic leaks.

**Fig. 2:**
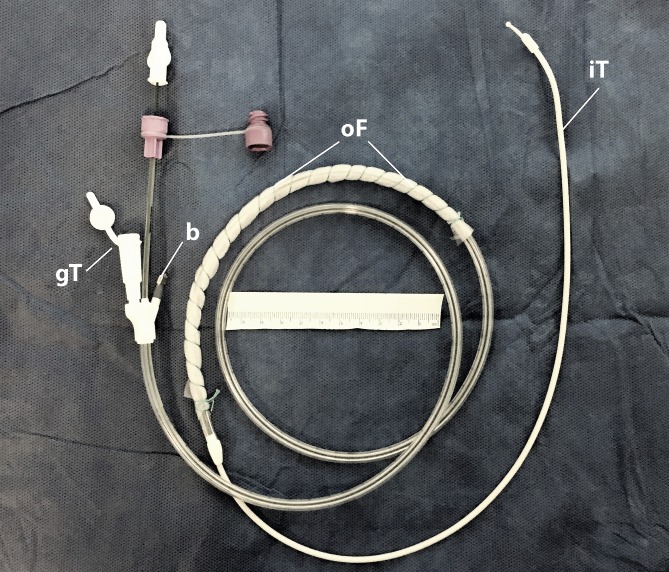
Double-lumen open-pore film drainage that can be used for active reflux drainage with simultaneous enteral feeding after abdominal-thoracic esophageal resection. The lateral perforation openings of the tube (Freka®Trelumina, Ch/Fri 16/9, 150 cm, Fresenius, Bad Homburg, Germany) are coated with the thin double-layered open-pore drainage film (*oF*; Suprasorb® CNP, Drainage Film, Lohmann & Rauscher International GmbH & Co, Rengsdorf, Germany). The ventilation tube is blocked with a clamp (*b*). Negative pressure is applied to the gastric tube opening (*gT*). *iT* Intestinal feeding tube

A further procedure to protect the anastomosis after esophageal resection is presently being investigated by our working group [37, 48]. A double-lumen OFD is placed in the stomach instead of a passive drainage tube (Fig. 2). Digestive juices, which may impair anastomotic wound healing, are actively and completely eliminated. The stomach is thus rendered dry for a few days after surgery and food is introduced via the integrated jejunal feeding tube.

A few years after the introduction of ENPT to treat anastomotic defects and perforations, there are now also prophylactic options and options for reflux prophylaxis to reduce surgical risk.

## ENPT in the duodenum

Endoscopic negative pressure therapy can also be used to treat duodenal leaks [49–56]. To reach the treatment site, surgical accesses (gastrostomy, jejunostomy) are also chosen [55, 56] and combined with new endoscopic techniques [19]. There were postoperative suture leaks in eight of ten patients; of these nine were treated with intraluminal ENPT. All the defects were healed after a median treatment duration of 11 days.

Bile is drained luminally from the defect by ENPT

Active drainage of bile by ENPT is crucial in the treatment of duodenal defects. Bile is drained luminally from the defect by ENPT. Contamination is stopped, and healing is promoted. Feisthammel et al. draw attention to the possible need for vitamin K substitution if nearly all the bile fluid is suctioned [53]. In the presence of an enterocutaneous fistula, the pull-through technique is a useful placement procedure, which considerably simplifies the introduction and replacement of the drain [52, 55].

Hochberger et al. have used intraluminal ENPT as active duodenal drainage to reduce the risk of perforation after removal of extensive duodenal polyps [57].

## ENPT in the pancreas

Anastomotic leakage after pancreaticogastrostomy has been successfully healed without the need for revision surgery [58, 59]. Active and complete intraluminal drainage of secretions also plays a very important role here [60]. Use of the pull-through technique to advance the drain along an enterocutaneous access point to the internal wound is also useful [32].

There are also isolated cases in which ENPT has been used to treat infected pancreatic cysts [38, 61, 62]. Wallstabe et al. have used this treatment with film-wrapped foam drains [38]; small-caliber OFDs also can be used to achieve the desired outcome [62].

## Combination with surgical procedures

ENPT in the upper gastrointestinal tract also has been combined with surgical procedures. Kühn et al. reported a revision rate of 40% in their patient population for esophageal leakages, whereby the actual local treatment of the defect occurred via ENPT. In other studies, the revision rate was only about 5%. Revision surgeries have become partially unnecessary by switching to the endoscopic procedure [63, 64].

For example, defects of the small intestine have been successfully healed in combination with surgeries using artificial access routes in the form of jejunal and gastric stomata [55, 56, 65].

## ENPT: a surgical endoscopic treatment procedure

The importance of this procedure lies particularly in the treatment of postoperative complications and specifically in intestinal anastomotic leakage. Anastomoses can be optimally inspected endoscopically at any time during and after surgery, defects can be diagnosed, and the internal wound status assessed.

Use of intraluminal negative pressure creates new options for surgical wound treatment within the body. This requires both extensive endoscopic expertise and experience in surgical wound healing. The wound status must be reassessed whenever the drain is changed, and the procedure individually adjusted accordingly.

## Practical conclusion

- ENPT has been adapted to treat defects in the upper gastrointestinal tract. The most extensive experience is in the treatment of transmural esophageal lesions.
- One possible indication might be the prophylactic use of negative pressure therapy after esophageal resection. However, this would require further investigation under study conditions. Likewise, the option of eliminating postoperative reflux may be evaluated.
- The importance of endoscopy in surgery has grown significantly with the introduction of ENPT and the resulting additional options.
